# Case report: a 5-year-old with new onset nephrotic syndrome in the setting of COVID-19 infection

**DOI:** 10.1186/s12882-021-02520-w

**Published:** 2021-09-27

**Authors:** Kelsi M. Morgan, Peace D. Imani

**Affiliations:** 1 Department of Pediatrics, Texas Children’s Hospital/ Baylor College of Medicine, 6621 Fannin St., Houston, TX 77030 USA; 2 Department of Pediatrics, Renal Section, Texas Children’s Hospital/ Baylor College of Medicine, Houston, USA

**Keywords:** Nephrotic syndrome, COVID-19, Proteinuria, And hypercoagulability

## Abstract

**Background:**

This is a case report of an asymptomatic SARS-CoV-2 infection associated with new-onset nephrotic syndrome in a pediatric patient. This is the third case of new-onset nephrotic syndrome in children associated with SARS-CoV-2 infection, but is the first case report describing a new-onset nephrotic syndrome presentation in a patient who had asymptomatic COVID-19 infection.

**Case presentation:**

This is a case of a previously healthy 5 year old female who presented with new-onset nephrotic syndrome in the setting of an asymptomatic COVID-19 infection. She presented with progressive edema, and laboratory findings were significant for proteinuria and hypercholesterolemia. She was treated with albumin, diuretics, and corticosteroid therapy, and achieved clinical remission of her nephrotic syndrome within 3 weeks of treatment. Though she was at risk of hypercoagulability due to her COVID-19 infection and nephrotic syndrome, she was not treated with anticoagulation, and did not develop any thrombotic events.

**Conclusions:**

Our case report indicates that SARS-CoV-2 infection could be a trigger for nephrotic syndrome, even in the absence of overt COVID-19 symptoms.

## Background

Since the outbreak of the COVID-19 pandemic, more than 20 million people in the United States alone have been infected with the SARS-Cov-2 virus, and over 350,000 of these people have died [1]. The symptoms and severity of illness vary widely among infected individuals with most impact on the respiratory, cardiovascular, kidney, hematologic, hepatic, cutaneous, gastrointestinal (GI) and nervous systems [2]. With regard to the pediatric population, as of December 31, 2020, just over 2.1 million cases of Covid-19 had been reported in children with an estimated 0.00–0.08% of all pediatric COVID-19 cases resulted in death [3]. Children thus seem to be infected with COVID-19 at a lower rate than adults, and tend to have less severe symptoms of disease. Despite this, there have been numerous reports of COVID-19 causing severe disease in children. Presentations of COVID-19 in children vary: the virus can cause severe symptoms upon initial infection, but children are also affected by multisystem inflammatory syndrome in children (MIS-C), which often occurs weeks after initial infection [4, 5]. Various presentations of MIS-C have included respiratory failure, myocardial dysfunction, hematological crises, gastrointestinal symptoms, among others [6].

Relevant to our case report are reported kidney complications especially studies in the adult population. A study by Yang et al. found that 15.4% of 91 adult patients with COVID-19 examined at autopsy had kidney injury at the time of death [7]. It is believed that kidney injury due to COVID-19 may be secondary to the ability of the virus to bind angiotensin converting enzyme 2 (ACE2) receptors, though the full mechanism may be more complex [8]. A recent study by Cheng et al. found that almost 44% of hospitalized adult patients had proteinuria, while a smaller number of patients (26.7%) had hematuria [9]. Proteinuria and collapsing glomerulopathy have also been described in patients with COVID-19 [10].

In children, kidney dysfunction in patients with COVID-19 has been reported, with AKI one of the most common kidney manifestations, both in absence and presence of MIS-C [11], [12]. Although the incidence of proteinuria in children with COVID-19 has not been well defined, there have been two case reports of new onset nephrotic syndrome in the setting of COVID-19 infection [13, 14]. In this report, we present another case of new-onset nephrotic syndrome in conjunction with positive COVID-19 testing.

## Case presentation

### Clinical presentation

A 5-year old, previously healthy female was admitted to the hospital in December 2020 with a 2-week history of periorbital swelling, with progressive involvement of abdominal and ankle swelling. Prior to admission, the child had developed isolated left eye swelling which was not associated with any other symptoms or vision changes. A week later, evaluation at her primary care physician (PCP)‘s office was notable for 4+ protein on urinalysis. The child was then referred to pediatric nephrology for further evaluation and management. In the renal clinic, on examination she was noted to have moderate abdominal swelling with ascites and pretibial edema. She had further gained 2 kg of weight since her visit to the pediatrician’s office 1 week prior. The child’s mother denied any recent illnesses such as fevers, chills, cough, rhinorrhea, congestion, nausea, vomiting, sore throat, gross hematuria, or diarrhea, and there were no known sick contacts. There was no known family history of kidney diseases.

### Laboratory findings

Urine analysis revealed 3+ proteinuria with no blood (Table 1). Spot urine protein was greater than 2 g/dL and urine protein-to-creatinine ratio greater than 12 mg/mg, which suggested the presence of nephrotic-range proteinuria. The patient had mild hyponatremia with serum sodium of 133 mmol/L. Serum creatinine was 0.27, and BUN was 20. Serum albumin was 2 g/dL, total cholesterol elevated at 307 mg/dL, elevated triglycerides at 644 mg/dL, with normal complements C3 and C4 at 87 mg/dL and 21 mg/dL, respectively. Our patient’s blood work was also notable for elevated D-dimer 2.49 (normal < 0.4 Ug/mL) and partial thromboplastin time (PTT) 39.1 (25.6–32.4) seconds. She had a mildly elevated TSH (5.6 IU/mL) with a normal free T4 (0.9 ng/dL), a low 25-hydroxy vitamin D level, and a low ferritin of 29. Of note, the surveillance COVID-19 testing (RT-PCR, performed via nasopharyngeal swab), which was performed as part of the general hospital admission process during the time of this case report, returned positive for SARS-CoV-2, and further immunoglobulin (Ig) testing was positive for both IgM and IgG antibodies. The SARS-CoV-2 RT-PCR nasopharyngeal swab test was performed using the Hologic Aptima SARS-CoV-2 Assay (approved for emergency use authorization by the US FDA), and the SARS-CoV-2 immunoglobulin testing was performed on the Abbott Architect i1000 platform (approved for emergency use authorization by the FDA).

**Table 1:** Laboratory parameters

Parameters	Initial clinic visit	5 weeks later	References
**Urine studies**			
Protein on dipstick	3+	negative	negative
Blood on dipstick	negative	negative	negative
Urine protein	> 2000		< 30 mg/dL
Urine protein-to-creatinine ratio	> 12	–	< 0.2 mg/mg
**Serum chemistry**			
Sodium	133	140	135–146 mmol/L
Potassium	4.4	4.3	3.8–5.1 mmol/L
Calcium	9.2^a^	9.9	8.8–10.1 mg/dL
Blood urea nitrogen	20	17	2–23 mg/dL
Creatinine	0.27	0.4	0.3–0.6 mg/dL
Albumin	2.0	4.1	3.5–4.7 g/dL
Complement C3	87	–	90–160 mg/dL
Complement C4	21	–	14–36 mg/dL
Vitamin D 25-hydroxy	6		30–80 ng/mL
**Lipid profile**			
Total cholesterol	307	–	112–208 mg/dL
Triglycerides	644	–	45–203 mg/dL
**Hematology**			
White blood cell count	7.12	13.2	4.86–13.38 × 10^3^/uL
Absolute neutrophil count	2790	4211	1500–8500 cells/uL
Absolute lymphocyte count	3620	7814	2000–8000 cells/uL
Hemoglobin	11.2	13.3	10.2–12.7 g/dL
Platelet count	371	431	189–403 × 10^3^/uL
**Coagulation profile**			
Partial thromboplastin time (PTT) (seconds)	39.1	32.1	
Fibrinogen (mg/dL)	510	270	
D Dimer (Ug/mL)	2.49	< 0.27	
**SARS-Cov-2 tests**			
SARS-Cov-2 RT-PCR^b^	Detected	–	
SARS-Cov-2 IgM	Positive	Positive	
SARS-Cov-2 IgG	Positive	Positive	

^a^Calcium corrected for hypoalbuminemia

^b^RT PCR real-time polymerase chain reaction

Table 1 Laboratory results for the patient at the time of diagnosis and 5 weeks later

### Clinical course and outcome

The patient received intravenous 25% albumin and furosemide for diuresis, with excellent response. In addition to a fluid and sodium restriction, she started on oral vitamin D supplements and following a negative purified protein derivative (PPD) skin test, she started corticosteroid therapy at 2 mg/kg per day. Throughout her four-day hospital stay, patient remained asymptomatic from COVID-19 perspective. Her vital signs were stable throughout admission (temperature: 36.1–36.9 C, pulse: 79–107 beats per minute, respiratory rate: 14–22 breaths per minute, SpO2: 97–100% on room air). Although our patient was at an increased risk for hypercoagulability from both nephrotic syndrome and COVID-19 infection, we did not initiate anticoagulation therapy. Patient was discharged home after 4 days at only 700 g (0.7 kg) above her baseline weight, as estimated by her recent weight at a PCP visit 2 months prior to admission.

Patient was in complete remission within 3 weeks of starting corticosteroids and urine protein was still negative after 6 weeks of therapy. Her coagulation profile and thyroid studies normalized without intervention; however, she remains positive for both IgM and IgG SARS-Cov-2 antibodies. Despite these positive results, she had still remained asymptomatic from a COVID-19 perspective at her follow up visit.

## Discussion

New-onset nephrotic syndrome following viral illnesses has been reported in literature (15) and SARS-Cov-2 infection may be one more of these viruses. There are two case reports of new-onset nephrotic syndrome in the setting of COVID-19 disease. We present the third case of new-onset nephrotic syndrome likely triggered by the novel SARS-Cov-2 infection.

In the two published case reports, both children had COVID-19-related symptoms prior to diagnosis of nephrotic syndrome. One of the cases is an eight-year old boy, with the typical age range for idiopathic nephrotic syndrome while the other is a 15-year old boy.

Our patient, however, developed nephrotic syndrome despite being otherwise asymptomatic from a COVID-19 perspective. This suggests that kidney involvement may be possible even in the absence of any other clear COVID-19 clinical symptoms.

An important consideration for our patient was whether or not to treat her with corticosteroids given her COVID-19 positive status, and the potential to worsen her infection. Previous case reports reported giving corticosteroids to patients with COVID-19 and new-onset nephrotic syndrome without negative outcomes, and the decision was thus made to treat our patient with conventional corticosteroid therapy [13, 14]. Our patient did well with this course of treatment, and did not have any overt clinical symptoms following initiation of corticosteroid therapy. We were interested to find that our patient continued to test positive for SARS-CoV-2 at her follow up visit 5 weeks after initial diagnosis. While we did not quantify the patient’s antibody titers, which makes it difficult to know whether and at what rate antibodies were declining at this repeat check, studies indicate that SARS-CoV-2 serum IgM begins to decline in the second month after onset of infection [16]. Thus, the continued presence of serum IgM 5 weeks after an initial positive test may be related to the timing of infection in our patient. At this time, it is not known whether or not steroids influence the rate of antibody decline, though this is perhaps something that could be studied by future researchers. In consonance with childhood nephrotic syndrome we elected not to proceed with kidney biopsy but initiate treatment with corticosteroids first [17]. Our patient did well, and had normalization of her coagulation profile following treatment of her nephrotic syndrome. She never required anticoagulation.

## Conclusion

This is the third case of new-onset nephrotic syndrome in the setting of COVID-19 in children. However, in contrast to previous reports, our patient was asymptomatic with COVID illness. It could be that SARS-Cov-2 virus could be the trigger for new onset nephrotic syndrome. The steroid responsiveness seen in majority of childhood nephrotic syndrome does not seem to be altered by SARS-Cov-2 infection. Need for anticoagulation should be assessed on a case-by-case basis. More studies are needed to understand the impact and long-term outcomes of COVID-19 on new-onset nephrotic syndrome in children. In absence of clinical trials, therapeutic guidelines become more apparent as more cases are reported.

## Data Availability

The data used for this case report is available upon reasonable request.

## Abbreviations

ACE2: Angiotensin converting enzyme 2
AKI: Acute kidney injury
COVID-19: Coronavirus 19
GI: Gastrointestinal
Ig: Immunoglobulin
MIS-C: Multisystem inflammatory syndrome in children
PCP: Primary care physician
PPD: Purified protein derivative
PTT: Partial thromboplastin time
RT-PCR: Reverse transcription-polymerase chain reaction
SARS-CoV-2: Severe acute respiratory syndrome coronavirus 2
